# Vitamin D deficiency in northern Taiwan: a community-based cohort study

**DOI:** 10.1186/s12889-019-6657-9

**Published:** 2019-03-22

**Authors:** Ming-Jse Lee, Heng-Jung Hsu, I-Wen Wu, Chiao-Yin Sun, Ming-Kuo Ting, Chin-Chan Lee

**Affiliations:** 1 Division of Nephrology, Chang Gung Memorial Hospital, 222 Mai-Chin Road, Keelung, 204 Taiwan; 2 College of Medicine, Chang Gung University, Tao-Yuan, Taiwan; 3 The Graduate Institute of Clinical Medical Sciences, Chang Gung University Medical College, School of Medicine, Taoyuan, Taiwan; 4 Division of Endocrinology, Chang Gung Memorial Hospital, Keelung, Taiwan

**Keywords:** Vitamin D deficiency, Prevalence, Risk factor, Taiwan

## Abstract

**Background:**

Vitamin D deficiency has become an important public health problem, however few studies have been conducted in subtropical countries, and the predictors of vitamin D deficiency in people with healthy renal function are unclear. The objective of this study was to evaluate the prevalence and factors associated with vitamin D deficiency in northern Taiwan.

**Methods:**

The cross-sectional study was performed between August 2013 and August 2017, and included 3954 participants without chronic kidney disease (CKD) aged ≥30 years in northern Taiwan. Serum 25-hydroxyvitamin D [25(OH)-D] levels, biochemistry, sociodemographic variables (age, sex, education, occupation) and lifestyle habits (tea, coffee consumption and physical activities) were recorded. Associations between vitamin D status and these variables were examined using a regression model. The definition of deficiency was defined as a serum 25(OH)-D level < 20 ng/mL (50 nmol/L).

**Results:**

The mean 25(OH)-D concentration was 28.9 ng/mL, and 22.4% of the study population had vitamin D deficiency. There was a significantly higher vitamin D deficiency ratio in the women compared to the men (22.9% vs 9.9%, *p* < 0.001). Vitamin D deficiency was most prevalent (38.4%) in those aged 30–39 years. Those with a graduate degree had the highest rate of vitamin D deficiency (31.5%). The predictors of vitamin D deficiency included female sex, young age, high education level, living in an urban area and physical inactivity. Tea consumption was negatively associated with vitamin D deficiency.

**Conclusions:**

Vitamin D deficiency is prevalent in subtropical areas such as northern Taiwan in healthy individuals without CKD.

## Background

The important role of vitamin D, a fat-soluble vitamin responsible for calcium and phosphate resorption, in bone health and mineralization is well known [1, 2]. Vitamin D deficiency may cause secondary hyperparathyroidism, rickets, osteomalacia, osteoporosis, and even fragility fractures [3]. In the past decade, vitamin D has also been shown to be involved in a wide variety of extra-skeletal effects, and its deficiency has been associated with several health conditions including muscle weakness [4], diabetes mellitus [5], chronic kidney disease [6], cancer [7, 8], cardiovascular disease [4], infection, and autoimmune disease [9].

Vitamin D can be obtained from sun light or natural food. However, natural food sources of vitamin D are limited and mainly come from animal food in the form of vitamin D3 only [10]. Thus, without artificial supplements or vitamin-fortified food, the major source of vitamin D comes from the action of ultraviolet-B light upon the 7-dehydrocholesterol of the skin. Several factors may influence the production of vitamin D in the skin, including aging, latitude, skin pigmentation, season, use of sun screen, outdoor activities and air pollution [11, 12].

Vitamin D deficiency has been reported to be more common than previously thought, and it has become a public health issue in modern societies [13, 14]. Many population-based studies on vitamin D deficiency have been conducted, however most have been performed in temperate countries with few being conducted in subtropical regions. Because the prevalence of vitamin D deficiency varies significantly in different countries and populations [15], investigating the prevalence and associated sociodemographic factors of vitamin D deficiency in subtropical areas is needed. In addition, to the best of our knowledge, no previous study has focused on healthy individuals without chronic kidney disease (CKD). Since the level of 25(OH)-D declines with renal function [16, 17], CKD may influence the results related to 25(OH)-D deficiency. In the present study, we evaluated the 25(OH)-D concentrations, lifestyle habits, exercise habits and past medical history, and also several demographic and laboratory variables from a large sample of individuals without CKD in Keelung, a northern city in Taiwan (latitude 25 N08’00″), to examine the prevalence and sociodemographic factors independently associated with 25 (OH) vitamin D [25(OH)-D] levels.

## Methods

### Study population and design

The study is based on data of a community health activity in four districts (Wanli, Ruifang, Gongliao and Anle) in northern Taiwan from August 2013 to August 2017. The community health activity included routine health examinations (including blood tests and urine analysis) and a questionnaire on health behavior for all residents in the community. The aim of this program was to detect and treat any health problems early and promote health. Residents of the four districts who aged ≥30 years and were not pregnant could join the health activity voluntarily after obtaining written informed consent. A total of 4925 participants joined the healthy activity and represent 4.2% of the population ages 30 and above in the four districts. All of the participants were enrolled. After excluding 971 participants with CKD, we obtained a cohort of 3954 participants. The participants were divided into two groups according to the level of plasma 25(OH)D; those with a level < 20 ng/mL (50 nmol/L) were considered to be vitamin D deficient [18, 19]. Demographic data (age, sex, residential district, occupation, and education level) and lifestyle habits (tea, coffee consumption and exercise) were assessed from the questionnaires. Anthropometric and biochemistry measurements were performed at entry to the study. Blood samples were obtained after an overnight fast, and the following parameters were determined: complete blood cell count, liver and renal biochemistry parameters, lipid profiles, fasting sugar, insulin, homeostatic model assessment of insulin resistance (HOMA IR), intact parathyroid hormone (iPTH) and total 25(OH)-D levels. This study was approved by the Ethics Committee of the Institutional Review Board of Keelung Chang Gung Memorial Hospital.

### Laboratory studies and definitions

We obtained complete laboratory profiles for individuals in both groups. The laboratory parameters included the plasma levels of blood urea nitrogen (BUN), creatinine, hemoglobin, albumin, high sensitive C reactive protein (hs-CRP), calcium, phosphate, alkaline phosphate, iPTH, hemoglobin A1C and cholesterol. Plasma levels of BUN, creatinine, hemoglobin, albumin, hs-CRP, calcium, phosphate, and cholesterol were assessed by spectrophotometric analysis using a modified kinetic Jaffe reaction with standardization of the creatinine calibration to an isotope dilution mass spectrometry reference measurement procedure. Plasma iPTH levels were measured using a commercially available radioimmunoassay kit (Scantibodies Laboratory; Santee, CA, USA). Serum level of 25(OH)-D was measured using an electro-chemiluminescence immunoassay (Cobas® Vitamin D3 assay, Roche Diagnostics GmbH, Mannheim, Germany) with an interassay coefficient of variation of 2.2–13.6%.

Chronic kidney disease was defined according to the National Kidney Foundation K/DOQI classification for CKD as persistent proteinuria or a decreased estimated glomerular filtration rate (eGFR) of < 60 mL/min/1.73 m^2^, determined using the abbreviated Modification of Diet in Renal Disease equation [20]. Proteinuria was defined as a urine albumin-to-creatinine ratio > 30 mg/g or urine protein-to-creatinine ratio > 150 mg/g. Vitamin D deficiency was defined as a 25(OH)-D level *<* 20 ng/mL (50 nmol/L). Body mass index (BMI) was calculated as the weight in kilograms divided by the square of the height in meters. The participants were defined as being tea and coffee drinkers if they had regularly drunk tea and coffee for > 5 years. The physical activity level was determined by weighting the reported hours per day of any physical activity such as walking, dancing, gardening, hiking, and swimming.

### Statistical methods

Demographic and anthropometric statistics were expressed as mean ± standard deviation as appropriate. The Student’s t-test was used to compare the means of continuous variables. Categorical data were tested using the Chi-square test. The prevalence of vitamin D deficiency was determined by sex, age group, education level, occupation, residential district, tea and coffee intake. Multiple logistic regression analysis was used to identify the independent predictors of vitamin D deficiency. Variables with a *P* value < 0.05 and tea consumption, which has been mentioned to have association with vitamin D deficiency, were included in the multiple logistic regression analysis. All reported *P* values were two-tailed, and were considered to be statistically significant if they were < 0.05. Data were analyzed using SPSS 17.0 for Windows (SPSS Inc., Chicago, IL).

## Results

A total of 3954 individuals without CKD aged ≥30 years were included in this study. The mean age of the study population was 55.48 ± 12.64 years. The mean 25(OH)-D concentration of the study group was 28.94 ± 10.27 ng/mL. Overall, 22.4% of the study population had a 25(OH)-D concentration < 20 ng/mL, and were defined as having vitamin D deficiency. Significantly more women had vitamin D deficiency than men (22.9% vs 9.9%, *P* < 0.001). The characteristics of the study group are presented in Table 1. The mean age of the normal vitamin D group was older than that of the vitamin D deficiency group (56.98 ± 12.18 vs 48.81 ± 12.53 years; *P* < 0.001). There were significantly more men in the normal vitamin D group than in the vitamin D deficiency group (38.8% vs 19.1; *P* < 0.001). In addition, the normal vitamin D group had a lower iPTH level (43.50 ± 19.10 vs 49.59 ± 22.69; *P* < 0.001) and higher hemoglobin level (13.91 ± 1.48 vs 13.26 ± 1.61; *P* < 0.001) than the vitamin D deficiency group. There were no significant differences in lipid profile, insulin and HOMR IR between the two groups.

**Table 1:** Baseline characteristics according to the presence or absence of vitamin D deficiency

	Normal	Vitamin D Deficiency	*p*-value
N	3230	724	
Age (years)	56.98 ± 12.18	48.81 ± 12.53	< 0.001*
Male (%)	1253/3230 (38.8)	138/724 (19.1)	< 0.001*
25(OH) D (ng/mL)	31.94 ± 8.77	15.49 ± 3.37	< 0.001*
Triglyceride (mg/dL)	118.50 ± 92.53	121.1 6 ± 110.50	0.500
HDL (mg/dL)	57.48 ± 14.98	56.67 ± 14.39	0.187
LDL (mg/dL)	126.90 ± 33.18	123.66 ± 31.94	0.099
Hemoglobin (g/dL)	13.91 ± 1.48	13.26 ± 1.61	< 0.001*
Alkaline P (U/L)	65.88 ± 19.56	63.38 ± 18.98	0.002*
Insulin (uU/mL)	7.91 ± 7.81	8.07 ± 7.25	0.630
HsCRP (mg/L)	2.15 ± 5.24	1.60 ± 2.43	< 0.001*
Creatinine (mg/dL)	0.74 ± 0.18	0.66 ± 0.15	< 0.001*
eGFR (ml/min/1.73 m2)	96.93 ± 21.52	107.93 ± 24.76	< 0.001*
Calcium (mg/dL)	9.36 ± 0.33	9.28 ± 0.33	< 0.001*
Phosphate (mg/dL)	3.81 ± 0.54	3.89 ± 0.51	< 0.001*
Albumin (g/dL)	4.71 ± 0.28	4.68 ± 0.27	0.011*
Hemoglobin A1C (%)	5.78 ± 0.68	5.73 ± 0.78	0.066
IPTH (pg/ml)	43.50 ± 19.10	49.59 ± 22.69	< 0.001*
HOMA IR (units)	2.10 ± 3.01	2.08 ± 2.52	0.915
BMI (kg/m2)	24.65 ± 3.72	23.96 ± 3.99	< 0.001*

Notes: Values are expressed as mean ± SD or total number (percent)

*p value < 0.05

Statistical significance based on the Chi-square test for categorical variables or t-test for continuous variables

Abbreviation: *HDL* high density lipoprotein-cholesterol, *LDL* low density lipoprotein-cholesterol, *Alkaline P alkaline phosphatase, HsCRP high sensitivity C-reactive protein*, *eGFR* estimate *glomerular filtration rate, IPTH intact parathyroid hormone*, *HOMA-IR* homeostasis model assessment of IR, *BMI* body mass index

**Fig. 1:**
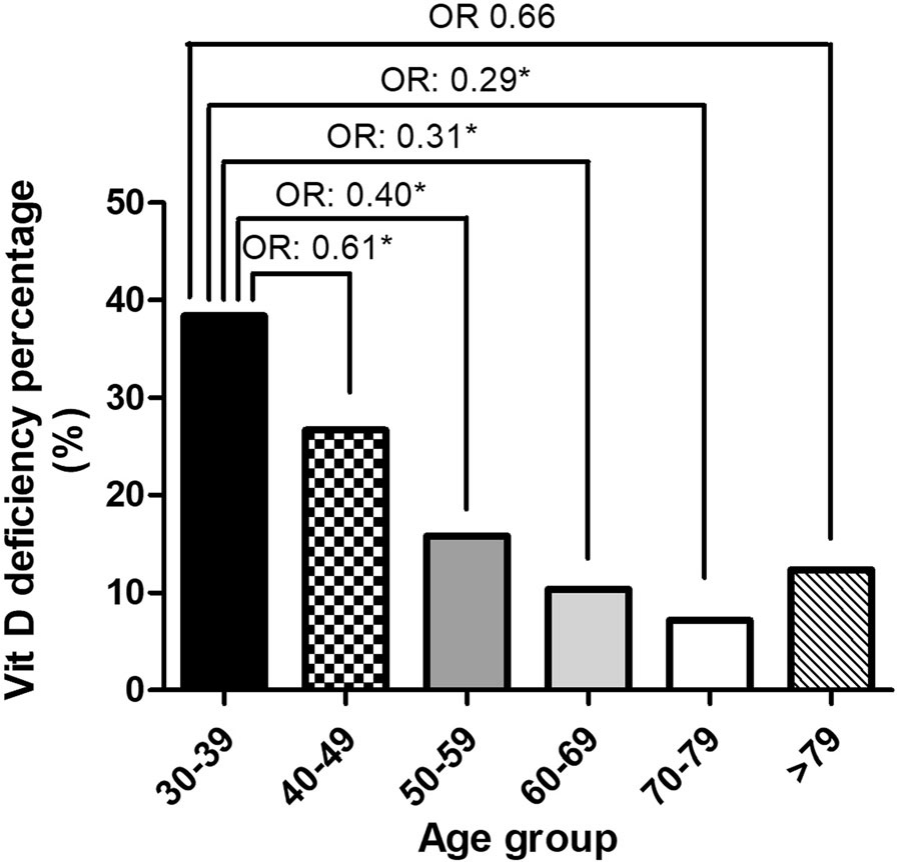
The prevalence of 25(OH) vitamin D deficiency in the study population in different age groups by multivariate analysis (*P < 0.05)

**Fig. 2:**
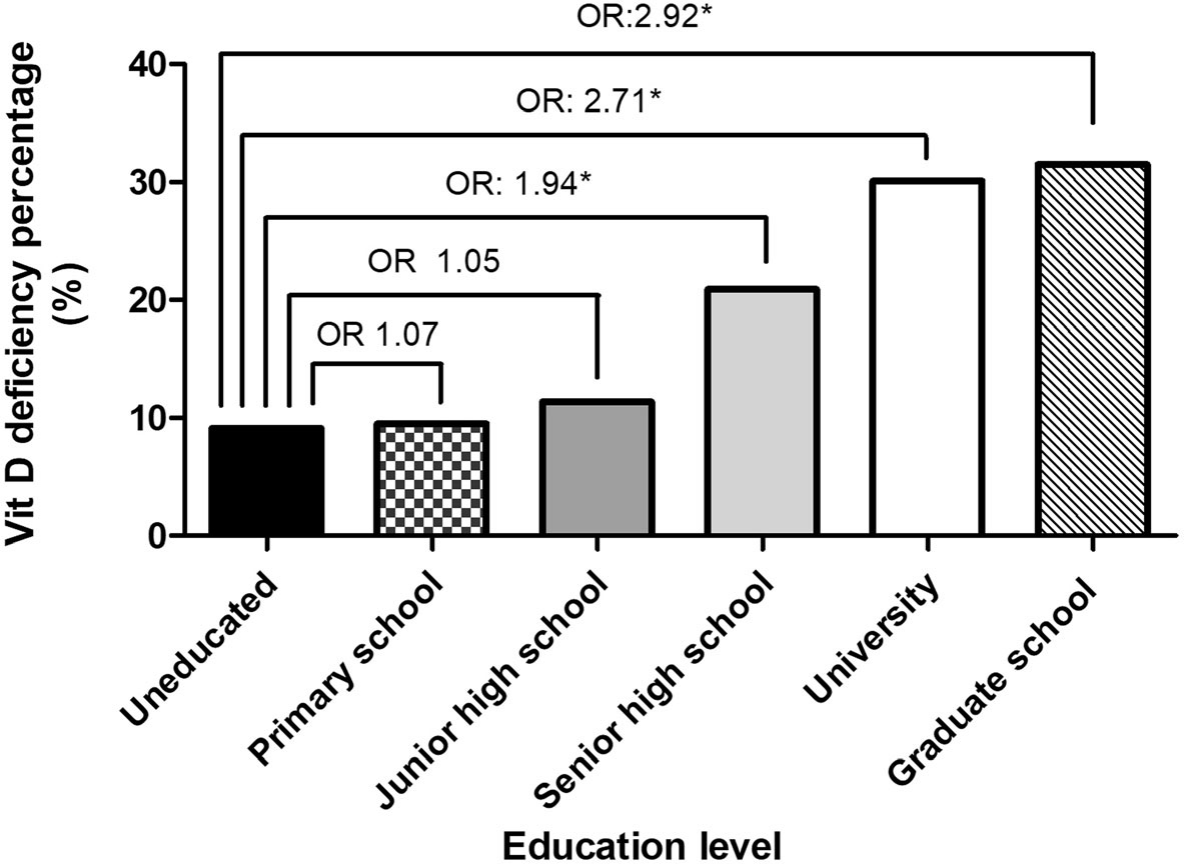
The prevalence of 25(OH) vitamin D deficiency in the study population in different education levels by multivariate analysis (*P < 0.05)

The prevalence of vitamin D deficiency in various age groups was illustrated in Fig. 1. The prevalence of vitamin D deficiency was highest in the participants aged 30 to 39 years (38.4%), and then decreased gradually after 40 years of age reaching the lowest level between 70 to 79 years of age (7.2%). However, the prevalence increased after 80 years of age (12.4%). The relationships between vitamin D deficiency and education level are demonstrated in Fig. 2. The ratios of vitamin D deficiency increased with increasing education level, with the highest rate observed in those with a graduate degree (31.5%).

**Fig. 3:**
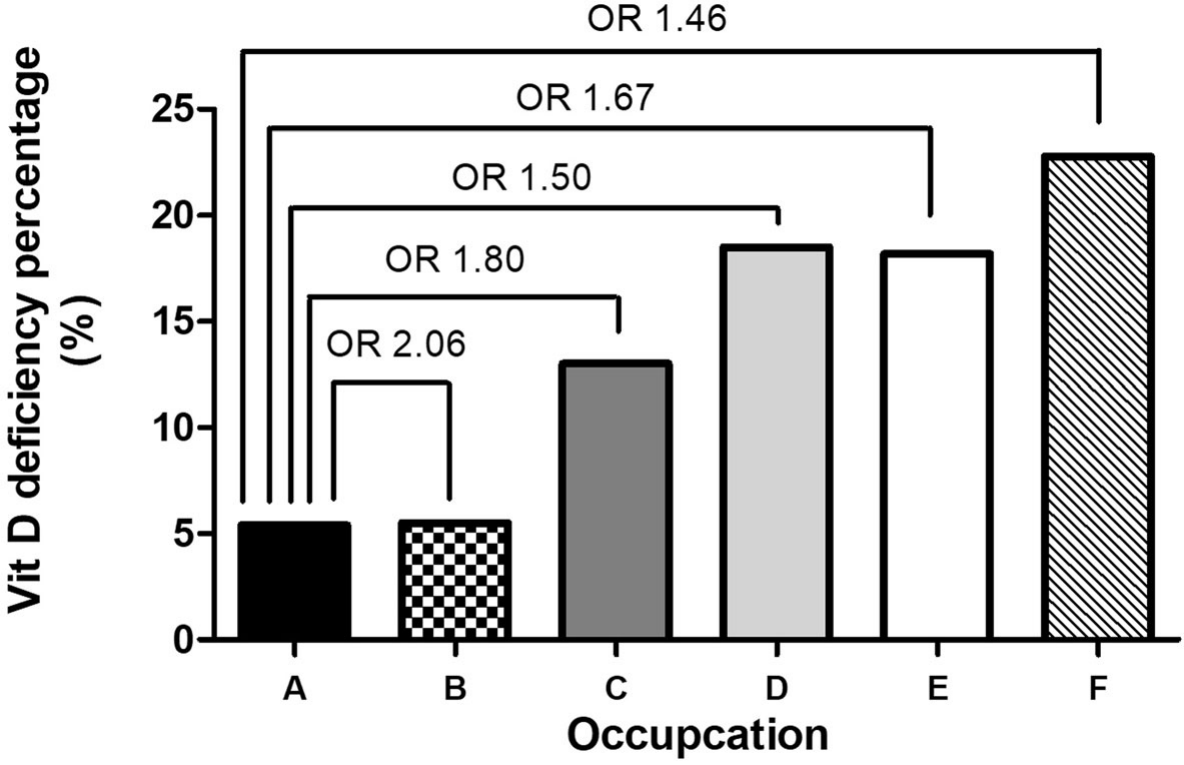
The prevalence of 25(OH) vitamin D deficiency in the study population in different occupations by multivariate analysis. Occupation classification: (**a**) (Agriculture); (**b**) (Fishery); (**c**) (Manufacturing Industry); (**d**) (Government employee); (**e**) (Homemaker); (**f**) (Service industry) (*P < 0.05)

**Fig. 4:**
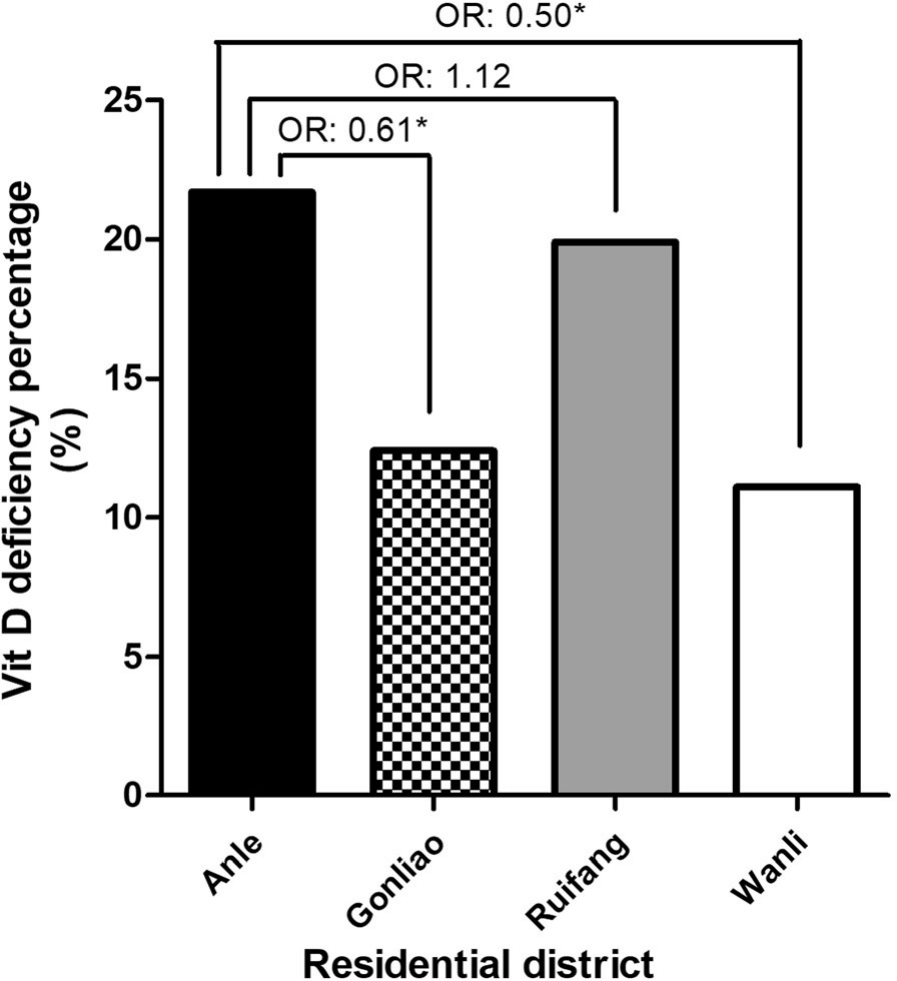
The prevalence of 25(OH) vitamin D deficiency in the study population in different residential districts by multivariate analysis (*P < 0.05)

The prevalence of vitamin D deficiency also varied by occupation (Fig. 3), with the lowest prevalence in farmers (5.4%) and the highest in service industry workers (22.8%). Overall, the individuals working in agriculture, fishery, and manufacturing had a lower prevalence of vitamin D deficiency than those working in the service industry, government employees, and homemakers (11.4% vs 21.1%, *P* < 0.001). Figure 4 shows the rates of vitamin D deficiency in the four study districts in northern Taiwan. Anle district had the highest percentage of vitamin D deficiency (21.7%), followed by Ruifang (19.9%), Gongliao (12.4%), and Wanli (11.1%) districts.

With regards to the effect of daily diet and behavior impacting the likelihood of vitamin D deficiency, regular coffee drinking was associated with a higher prevalence of vitamin D deficiency than non-consumption of coffee (20.9% vs 13.9, *P* < 0.001). In contrast, there was no significant difference in vitamin D deficiency between the individuals who did and did not regularly drink tea (19.0% vs 16.9%, *P* = 0.100) or take vitamin D supplements (23.6% vs 18.1%, *P* = 0.101). In addition, increased physical activity reduced the likelihood of developing vitamin D deficiency.

In univariate analysis, many factors were associated with vitamin D deficiency, so we performed multiple logistic regression analysis including all factors which showed that younger age (30–40, 40–50, 50–60, 60–70 years), female sex, higher education level (graduate school, university, senior high school), less physical activity, and urban residential area (Anle district) were significantly independently associated with vitamin D deficiency, and that tea consumption was negatively independently associated with vitamin D deficiency (Table 2).

**Table 2:** Multiple logistic regression analysis for associations with vitamin D deficiency

	Univariate Odds ratio	95% CI	*P* value	Multivariate Odds ratio	95% CI	*P* value
Male	0.372	0.305–0.453	< 0.001*	0.339	0.269–0.429	< 0.001*
Age (years)						
30–40			Ref.			Ref.
40–50	0.585	0.458–0.747	< 0.001*	0.605	0.460–0.795	< 0.001*
50–60	0.302	0.240–0.381	< 0.001*	0.396	0.301–0.520	< 0.001*
60–70	0.186	0.144–0.241	< 0.001*	0.309	0.223–0.430	< 0.001*
70–80	0.124	0.083–0.187	< 0.001*	0.292	0.176–0.484	< 0.001*
> 80	0.227	0.118–0.436	< 0.001*	0.662	0.299–1.468	0.308
Occupation						
Agriculture			Ref.			Ref.
Fishery	1.019	0.266–3.905	0.978	1.460	0.358–5.951	0.597
Manufacturing industry	2.637	0.810–0.584	0.107	1.666	0.477–5.824	0.424
Government employee	4.023	1.223–13.226	0.022*	1.496	0.420–5.328	0.535
Homemaker	3.936	1.211–12.801	0.023*	1.801	0.516–6.289	0.357
Service industry	5.226	1.624–16.817	0.006*	2.058	0.594–7.132	0.255
Education level						
Un-educated			Ref.			Ref.
Primary school	1.050	0.653–1.690	0.840	1.065	0.619–1.832	0.820
Junior high school	1.276	0.784–2.077	0.326	1.051	0.586–1.884	0.868
Senior high school	2.629	1.687–4.098	< 0.001*	1.944	1.111–3.400	0.020*
University	4.298	2.758–6.698	< 0.001*	2.706	1.510–4.848	0.001*
Graduate school	4.575	2.552–8.204	< 0.001*	2.915	1.390–6.115	0.005*
Residential district						
Anle			Ref.			Ref.
Gongliao	0.509	0.386–0.670	< 0.001*	0.608	0.439–0.843	0.003*
Ruifang	0.894	0.719–1.113	0.317	1.122	0.869–1.450	0.377
Wanli	0.451	0.348–0.585	< 0.001*	0.500	0.373–0.670	< 0.001*
Coffee (yes)	1.635	1.368–1.955	< 0.001*	1.170	0.943–1.453	0.154
Tea (yes)	1.155	0.973–1.372	0.100	0.755	0.612–0.933	0.009*
Physical activity (hours/day)	0.499	0.410–0.607	< 0.001*	0.644	0.522–0.795	< 0.001*

*p value < 0.05.

Statistical significance based on the Chi-square test for categorical variable

## Discussion

This study examined 25(OH)-D levels, the prevalence of vitamin D deficiency and the associated predictors in healthy adults with normal renal function in northern Taiwan. To the best of our knowledge, this is the first study to focus on a large sample of individuals without CKD. Overall, we found that vitamin D deficiency was common even in this population, and that the prevalence was particularly high in women, those with a younger age, those who were better educated, and those who lived in an urban area. In addition, tea consumption seemed to be a protective factor against vitamin D deficiency.

In the present study, the mean 25(OH)-D concentration of the study group was 28.94 ± 10.27 ng/mL and 22.4% had vitamin D deficiency (25(OH)-D concentration < 20 ng/mL). In the population-based National Health and Nutrition Examination Survey conducted in the United States from 2001 to 2006, 32% of the population had a serum 25(OH)-D concentration < 20 ng/mL [21]. In addition, the Korea National Health and Nutrition Examination Survey conducted in 2008 reported prevalence rates of vitamin D deficiency (< 20 ng/mL) of 47.3% in males and 64.5% in females [22]. In contrast, in a nationwide population-based study conducted in Thailand, only 5.7% of the population had a 25(OH)-D level < 20 ng/mL [23]. The prevalence rate of vitamin D deficiency in the current study was lower than those in the studies from the United States and Korea but higher than that in the study from Thailand, which may reflect the effect of latitude. As sun exposure is an important factor for vitamin D synthesis, people living at a lower latitude may have more sun exposure and therefore a lower prevalence of vitamin D deficiency.

Many studies have demonstrated an increasing prevalence of vitamin D deficiency with age [24–26]. The main reason may be that the elderly have decreased concentrations of 7-dehydrocholesterol, the precursor of vitamin D3, and therefore have a decreased ability to make vitamin D in the skin [27]. However, in the current study, vitamin D deficiency was less prevalent with advancing age. Moreover, a young age was a risk factor for vitamin D deficiency. Some studies have reported that the elderly use more vitamin D supplements, and this may explain the higher vitamin D value in the elderly [28]. However, we found that the elderly subjects in this study took less vitamin D supplements than the younger subjects (30–39 years old: 34.6%, 40–49 years old: 34.8%, 50–59 years old: 32.1%, 60–69 years old: 27.5%, 70–79 years old: 16.8%, > 79 years old: 15.7%; *P* < 0.001). Therefore, other factors must contribute to this phenomenon. The amount of sun exposure is a possible factor. Young people tend to spend more time indoors for study or work in Taiwan. In contrast, the elderly may be able to spend more time outdoors [22, 23]. Moreover, young people may use more sunblock because of cosmetic issues, and therefore have less exposure to the sun [23].

We also investigated the effect of residential district on vitamin D level, and found that Anle district, which is an urban area, had a higher proportion of vitamin D deficiency than Gongliao and Wanli districts, which are rural areas. This finding is consistent with many other studies [23]. There are several reasons that may explain the higher proportion of vitamin D deficiency in an urban area. First, people in urban areas tend to spend more time indoors due to their jobs and lifestyle. Second, air pollution may be a risk factor for vitamin D deficiency, and urban inhabitants are exposed to higher levels of air pollution than rural inhabitants [11]. However, according to data from the Central Weather Bureau of Taiwan, the concentrations of ozone and fine particulate matter (PM 2.5) are lower in urban areas than rural areas. Therefore, lifestyle factors may be the reason why the residents of the urban area had a higher percentage of vitamin D deficiency than those in rural areas in the present study.

Occupation was an important determinant of vitamin D deficiency in this study, and those who worked indoors (including government employees, homemakers, and service industry workers) had a higher risk of vitamin D deficiency than those who worked outdoors (including agriculture and fishery workers). Education levels also had a significant impact on vitamin D deficiency, and the subjects with a higher education level were associated with a higher risk of vitamin D deficiency. This finding is similar to the report by Daly et al. [25]. However, in the multiple logistic regression analysis including all factors, education level remained a risk factor of vitamin D deficiency whereas occupation did not. This may reflect that the effect of education level was stronger than that of occupation. Education level would influence the choice of occupation, lifestyle and behavior factors. Those with a higher education level tend to be younger, have indoor jobs, and be more concerned about skin whitening and sun protection [29, 30]. In contrast, those with a lower education level tend to be older, have outdoor jobs and not care about sun protection.

We also found a relationship between vitamin D deficiency and tea and coffee consumption. To the best of our knowledge, few studies have investigated this relationship [31]. Although there was no significant difference in the prevalence of vitamin D deficiency between those who did and did not consume tea, tea consumption appeared to be a protective factor against vitamin D deficiency after multiple regression analysis adjusting for confounding variables such as age, education and residential districts. It is likely that these factors may confound the association between vitamin D deficiency and tea consumption. About the influence of age on the association of vitamin D deficiency and tea consumption, the analysis showed that the people who consumed tea were younger than those who did not consume tea (53.05 ± 12.10 vs 59.73 ± 12.50 years; *P* < 0.001). The younger participants had a higher prevalence of vitamin D deficiency, which may have masked the benefit of tea consumption with regards to 25(OH)-D level. In contrast, coffee consumption was associated with a higher prevalence of vitamin D deficiency compared to no coffee consumption. However, coffee consumption was not an independent risk factor for vitamin D deficiency after multiple logistic regression analysis, which is consistent with the findings of Al-Othman A et al. [31]. The mechanism underlying the positive effect of tea consumption on the 25(OH)-D level is not entirely clear, and further studies are needed to clarify this relationship.

The strengths of this study are the large study population and excluding patients with CKD. However, there are some limitations to the present study. First, we did not obtain information about dietary intake of vitamin D, the amount of sun exposure and other factors that may have influenced sun exposure, such as clothing, the amount of time spent outdoors, the use of sun-screen, and skin color. All of these factors could affect the 25(OH)-D level. Second, some of the data such as exercise and tea/coffee consumption were obtained from questionnaires, which may have introduced reporting or recall bias. Third, we did not estimate the effect of the season or month of blood sample collection on vitamin D deficiency. Fourth, the method we used to measure 25(OH)-D values (radioimmunoassay) may have resulted in lower values than the gold standard (liquid chromatography tandem mass spectrometry), and may have overestimated the prevalence of vitamin D deficiency [32]. Finally, our data were cross-sectional, and thus we could not analyze longitudinal changes in vitamin D.

## Conclusions

In conclusion, our data demonstrated that vitamin D deficiency is prevalent in northern Taiwan, even in healthy individuals without CKD. The prevalence was particularly high in women, those who were younger, better educated, and who lived in an urban area. Vitamin D supplements are thus an important issue in this group of people. Furthermore, we also found that tea consumption had a protective effect on vitamin D deficiency. Further studies are needed to confirm our findings.
